# Prevalence, Clinical Staging and Risk for Blood-Borne Transmission of Chagas Disease among Latin American Migrants in Geneva, Switzerland

**DOI:** 10.1371/journal.pntd.0000592

**Published:** 2010-02-02

**Authors:** Yves Jackson, Laurent Gétaz, Hans Wolff, Marylise Holst, Anne Mauris, Aglaé Tardin, Juan Sztajzel, Valérie Besse, Louis Loutan, Jean-Michel Gaspoz, Jean Jannin, Pedro Albajar Vinas, Alejandro Luquetti, François Chappuis

**Affiliations:** 1 Division of Primary Care Medicine, Department of Community Medicine And Primary Care, University Hospitals of Geneva and Faculty of Medicine, Geneva University, Geneva, Switzerland; 2 Division of Humanitarian and International Medicine, Department of Community Medicine and Primary Care, University Hospitals of Geneva and Faculty of Medicine, Geneva University, Geneva, Switzerland; 3 Department of Genetics and Laboratory, University Hospitals of Geneva and Faculty of Medicine, Geneva University, Geneva, Switzerland; 4 Division of Cardiology, Department of General Internal Medicine, University Hospitals of Geneva and Faculty of Medicine, Geneva University, Geneva, Switzerland; 5 Department of Radiology, University Hospitals of Geneva and Faculty of Medicine, Geneva University, Geneva, Switzerland; 6 Control of Neglected Tropical Diseases, World Health Organization, Geneva, Switzerland; 7 Laboratorio de Chagas, Hospital das Clinicas, Universidade Federal de Goiás, Goiania, Brazil; Institute of Tropical Medicine, Belgium

## Abstract

**Background:**

Migration of Latin Americans to the USA, Canada and Europe has modified Chagas disease distribution, but data on imported cases and on risks of local transmission remain scarce. We assessed the prevalence and risk factors for Chagas disease, staged the disease and evaluated attitudes towards blood transfusion and organ transplant among Latin American migrants in Geneva, Switzerland.

**Methodology/Principal Findings:**

This cross-sectional study included all consecutive Latin American migrants seeking medical care at a primary care facility or attending two Latino churches. After completing a questionnaire, they were screened for Chagas disease with two serological tests (Biomérieux ELISA cruzi; Biokit Bioelisa Chagas). Infected subjects underwent a complete medical work-up. Predictive factors for infection were assessed by univariate and multivariate logistic regression analysis.1012 persons (females: 83%; mean age: 37.2 [SD 11.3] years, Bolivians: 48% [n = 485]) were recruited. 96% had no residency permit. Chagas disease was diagnosed with two positive serological tests in 130 patients (12.8%; 95%CI 10.8%–14.9%), including 127 Bolivians (26.2%; 95%CI 22.3%–30.1%). All patients were in the chronic phase, including 11.3% with cardiac and 0.8% with digestive complications. Predictive factors for infection were Bolivian origin (OR 33.2; 95%CI 7.5–147.5), reported maternal infection with *T. cruzi* (OR 6.9; 95%CI 1.9–24.3), and age older than 35 years (OR 6.7; 95%CI 2.4–18.8). While 22 (16.9%) infected subjects had already donated blood, 24 (18.5%) and 34 (26.2%) considered donating blood and organs outside Latin America, respectively.

**Conclusions:**

Chagas disease is highly prevalent among Bolivian migrants in Switzerland. Chronic cardiac and digestive complications were substantial. Screening of individuals at risk should be implemented in nonendemic countries and must include undocumented migrants.

## Author Summary

Chagas disease, a parasitic disease caused by *Trypanosoma cruzi*, is a leading cause of cardiac and digestive tract disorders in Mexico, Central and South America. An increasing number of cases have recently been reported in North America and Europe due to international human migration, but data outside Latin America remains scarce. This study showed that Chagas disease is an emerging health problem in Switzerland, affecting a substantial proportion of Latin American migrants (13%). Persons at increased risk of infection were Bolivian, older than 35 years or had a mother infected with *T. cruzi*. Early signs of cardiac or digestive tract disease were found in one out of six infected patients. The risk of local transmission by blood transfusion or organ transplant was illustrated by the frequent willingness expressed by patients to donate blood or organs in Switzerland. The authors recommend the screening of persons at risk of infection and the diffusion of appropriate information to the medical community to increase awareness of this emerging health problem. Considering that affected persons frequently lack health insurance in Switzerland, a facilitated access to medical care is an important step towards better recognition and management of Chagas disease.

## Introduction

Chagas disease is a zoonosis caused by *Trypanosoma cruzi* (*T. cruzi*), a flagellated protozoa transmitted to humans by the faeces of blood-sucking triatomine bugs. The parasite can also be acquired by blood transfusion, organ transplant, ingestion of food contaminated with triatomines or their feces, or congenital transmission [1]. In 2009, we celebrate the 100^th^ anniversary of the first complete description of the disease by Carlos Chagas, a Brazilian physician. Chagas disease affects eight to ten million people worldwide and kills more than any other parasitic disease in Latin America [2]. Until recently, its geographical distribution was mostly determined by the area of endemicity of the infected vectors. Successful vector control in endemic countries, urbanization, human migration and unpreparedness of newly affected areas have contributed to modify the distribution of Chagas disease [3],[4]. Non-endemic countries (i.e. countries free of vectors) in North America, Europe and Western Pacific Region have seen the recent emergence of Chagas disease following the migration of more than 15 million people from endemic areas [4]. Estimates of the total number of *T. cruzi* infected people living in non-endemic countries and reported cases are both on the rise, reaching an estimated 25'000 to 40'000 in Western European countries in 2008. [4]–[11]. These estimates are usually based on the number of registered Latin American migrants in the recipient country multiplied by the mean prevalence of the disease among blood donors in the home country. This mode of calculation has several limitations, as it does not include migrants without legal registration (undocumented), and does not take into account regional variations of disease prevalence within endemic countries [12].

Acute *T. cruzi* infection - frequently asymptomatic - is followed by a long period of latency with few or no circulating parasites (indeterminate form of the chronic phase) [1]. After decades, 20–30% of infected persons develop chronic cardiac or digestive tract complications. Chronic chagasic cardiopathy (CCC), which is responsible for the high morbidity, mortality, and socio-economical impact of the disease in affected areas, is frequently underdiagnosed, particularly in non-endemic countries [7]. ECG is the recommended screening test for cardiac damage [13]. Up to 10% of *T. cruzi* infected persons may develop gastro-intestinal motility disorder leading to progressive dilatation of the oesophagus (megaoesophagus) and/or the colon (megacolon). Suspected digestive tract complications are investigated by barium studies. Specific clinical features of Chagas disease in non-endemic countries are not well characterized and may differ from those found in Latin America for various reasons, including different duration of exposure to infection. Until now, the few published studies describing disease patterns in Europe were conducted in specialized centres, and the findings may not be extrapolated to the global Latin American migrant community [5],[14].

Blood-borne transmission of *T.cruzi* has been reported in several non-endemic countries [11],[15]. Prevalence of infected blood donations in Europe and North America varies widely, reaching 0.62% in at-risk donors in Spain [15]. Recently, USA, Spain and France have implemented measures to reduce transfusional risk through blood donors screening and deferral strategies [11]. However, most European countries that may harbour blood donors at risk have yet to implement screening measures. The attitude and practice of Latin American migrants towards blood donation in non-endemic countries has yet to be investigated.

In 2008, Switzerland hosted 43'000 legal residents originating from Central and South America [16]. This figure did not include Swiss nationals from Latin American origin and the estimated 30–50'000 undocumented Latin American migrants. Undocumented migrants experience difficulties in accessing medical care in Switzerland, as health insurance is mandatory and expensive [17]. The first report of Chagas disease in Switzerland goes back to 1996. Since then, several imported and congenital cases have been reported [18],[19].

The objectives of this descriptive transversal study were to (1) determine the prevalence of Chagas disease in a community of Latin American adult migrants living in Geneva, (2) assess the risk factors for *T. cruzi* infection, (3) clinically stage the disease, and (4) evaluate the transfusional and transplantational risk to local recipients.

## Methods

### Setting

The study took place in a primary care centre (the Community Care Mobile Unit) of the Geneva University Hospitals which provides affordable care to more than 2000 Latin American migrants yearly, the majority of them living in Geneva with neither residency permit nor health insurance. Privacy is strictly ensured for undocumented persons. This unit cooperates closely with representatives of migrants communities. Information about the study was widely diffused in cultural centres, churches and migrant associations. In addition, two recruitment sessions took place in churches attended by migrants.

### Participants and procedures

Between June and December 2008, all consecutive adult Latin American migrants were invited to participate to the study. Other inclusion criteria were age more than 16 years and signature of an informed consent form. Pregnant women were excluded from the study and were referred to the Maternity ward of the Geneva University Hospitals where a specific program for Chagas screening has been ongoing since January 2008 [9]. Written informed consent was requested from participants. Participants completed a questionnaire (available in Spanish, Portuguese and French) that collected socio-demographic and medical data, and assessed their prior and current attitudes towards blood donation and organ transplant. A multilingual volunteer was available to help onsite. Serological tests, clinical investigations and treatment were free of charge. This study was approved by the ethics committee of the Geneva University Hospitals in January 2008 (protocol 07-285).

### Diagnosis of *T. cruzi* infection

Peripheral blood was drawn by a qualified nurse and serum was kept refrigerated at −20°C. Two commercialized ELISA-based serological tests (ELISA cruzi, Biomérieux, Brazil and Bioelisa Chagas, Biokit, Spain), which detect antibodies against crude and recombinant *T. cruzi* antigens respectively, were performed according to manufacturers' instructions with Dynatech-MRW Microplate Washer. Chagas disease was diagnosed when both tests were positive. The two tests were repeated in case of discrepant results (e.g. positive-negative; doubtful-negative). External quality control consisted of testing serum samples from all individuals with positive or discordant ELISA tests and from 10% of individuals with negative tests (Laboratory of Chagas disease, Goias University, Brazil). A combination of four serological tests was performed (Chagatek ELISA, Biomérieux, Argentina; EIE Chagas Bio-Manguinhos, Brasil; Chagatest HAI, Wiener, Argentina; in-house immunofluorescent test using Biomerieux conjugate, Biomérieux, Brazil). Results were sent back with an integrated conclusion (positive, negative or non-conclusive).

### Staging and management of the disease

All individuals with confirmed *T. cruzi* infection were contacted by phone and underwent a clinical evaluation that included full medical history, physical examination, and a 12-lead electrocardiogram (ECG) with a 30-second DII strip. In case of symptoms or signs suggestive of cardiac failure, history of syncope, or ECG changes consistent with CCC, an echocardiogram and a 24-hour Holter recording were performed. Results of cardiac investigations were independently reviewed by two cardiologists. The classification of CCC was based on the Brazilian Consensus [20]. Patients with dysphagia to solid or liquid food and/or with severe constipation (less than 2 stools per week and/or use of laxatives more than 5 days per week for more than 6 months) underwent gastro-intestinal tract barium examination. Oesophageal abnormalities were staged according to the classification of de Rezende [21]. The colon was considered abnormal if its diameter exceeded 6cm. In the absence of abnormal findings by ECG, echocardiography, 24-hour Holter recording, and barium studies, Chagas disease was classified in the indeterminate form of the chronic phase. According to recent recommendations, all eligible cases were treated with nifurtimox or benznidazole for 60 days [13].

### Statistical analysis

In order to investigate the relationship between Chagas disease and possible predictive factors, we used 2×2 tables and performed Chi-square and Fisher's exact tests for categorical variables and unpaired Student's t-tests for continuous variables. Univariate and multivariate logistic regression analyses were used to assess factors associated with Chagas disease. All analyses were performed using SPSS for Windows (version 15.0).

## Results

Thousand-and-twelve participants, with a mean age of 37.2 (standard deviation (SD) 11.3) years and a female predominance (83%), were recruited in the study. Ninety-six percent of participants were undocumented. Countries of origin were Bolivia (n = 485; 48%), Brazil (n = 249; 25%), Colombia (n = 61; 6%), Peru (n = 58; 6%), Ecuador (n = 47; 5%), Paraguay (n = 28; 3%), Nicaragua (n = 24; 2%), Honduras (n = 24; 2%) and others (n = 36; 4%). The mean duration of stay outside Latin America was 4.9 (SD 4.0) years. Socio-demographic characteristics and medical history data related to *T. cruzi* infection of the 1012 participants are shown in Table 1. Previous bite by triatomine bugs were reported by 11.2% of the participants, 12.7% had received blood transfusion in Latin America and 7.7% were born from a *T. cruzi* infected mother. Previous positive testing for Chagas disease was reported by 2.6% of participants.

**Table 1:** Description of Latin American migrants (n = 1012) with and without Chagas disease (CD) in Geneva, Switzerland.

	Total population N = 1012, Mean (SD) or n (%)	Subjects with CD, N = 130, Mean (SD) or n (% within CD)	Subjects without CD, N = 882, Mean (SD) or n (% without CD)	p*
**Age (mean)**	37.4 (SD 11.3)	41.0 (SD 9.4)	36.9 (SD 11.5)	<0.0001
**Age in 2 groups**				<0.0001
≤35 years	494 (48.8%)	37 (28.5%)	457 (51.8%)	
>35 years	518 (51.2%)	93 (71.5%)	425 (48.2%)	
Sex (female)	835 (82.5%)	108 (83.1%)	727 (82.4%)	0.85
**Origin**				<0.0001
Bolivia	486 (47.5%)	127 (97.7%)	359 (40.7%)	
other	528 (52.5%)	3 (2.3%)	523 (59.3%)	
**Years outside Latin America**	4.9 (SD 4.0)	4.6 (SD 2.1)	4.9 (SD 4.2)	0.41
**Mother with ** ***T. cruzi*** ** infection**				<0.0001
Yes	78 (7.7%)	26 (20.0%)	52 (5.9%)	
No	604 (59.7%)	47 (36.2%)	557 (63.2%)	
Don't know	330 (32.6%)	57 (43.8%)	273 (31.0%)	
**Previous testing**				<0.0001
Yes	80 (7.9%)	27 (20.7%)	53 (6.1%)	
No	857 (84.7%)	95 (73.1%)	762 (86.4%)	
Don't know	75 (7.4%)	8 (6.2%)	67 (7.6%)	
**Previous triatomine bite**				<0.0001
Yes	113 (11.2%)	35 (26.9%)	78 (8.8%)	
Now	336 (33.2%)	20 (15.4%)	316 (35.8%)	
Don't know	563 (55.6%)	75 (57.7%)	488 (55.3%)	
**Previous transfusion**				0.001
Yes	129 (12.7%)	30 (23.1%)	99 (11.2%)	
No	879 (86.9%)	100 (76.9%)	779 (88.3%)	
Don't know	4 (0.4%)	0	4 (0.5%)	
**Previous treatment**				0.002
Yes	10 (1.0%)	5 (3.8%)	5 (0.6%)	
No	1001 (98.9%)	125 (96.2%)	876 99.3%)	
Don't know	1 (0.1%)	0	1 (0.1%)	

*p concerns difference between subjects with and without Chagas disease.

### Prevalence of Chagas disease and predictive factors

On the basis of concordant positive serological tests, *Trypanosoma cruzi* infection was diagnosed in 130 participants, resulting in an overall prevalence of 12.8% (95% confidence interval (CI) 10.8–14.9); prevalence among Bolivians was 26.2% (95%CI 22.3–30.1; n = 127). External quality control confirmed all positive cases. Three infected individuals originated from Argentina (n = 2) and Brazil (n = 1); all had lived for several years in Bolivia. All (n = 12) discordant serological results in Geneva were controlled at the reference laboratory and proved to be negative. Socio-demographic characteristics and clinical data of *T. cruzi* infected individuals compared to non-infected ones and analysis of factors associated with infection are shown in Tables 1 and 2, respectively. Multivariate analysis showed that major predictive factors for *T. cruzi* infection were Bolivian origin (adjusted odds ratio (OR 33.2; 95%CI 7.5–147.5), maternal infection with *T. cruzi* (OR 6.9; 95%CI 1.9–24.3), and age older than 35 years (OR 6.7; 95%CI 2.4–18.8).

**Table 2:** Predictive factors, unadjusted and adjusted odds ratios (OR) for Chagas disease (CD) among *T. cruzi* infected Latin American migrants in Geneva, Switzerland (n = 130).

	Prevalence of CD, n (%)	Unadjusted OR for CD (95%CI)	Adjusted OR for CD° (95%CI)	Adjusted OR for CD* (95%CI)
**Age (years)**				
≤35	37/494 (7.5%)			
>35	93/518 (18.0%)	2.7 (1.8;4.0)	3.6 (2.3;5.6)	6.1 (2.2;16.7)
**Gender**				
Women	108/835 (12.9%)	1.04 (0.6;1.7)	0.85 (0.5;1.5)	1.04 (0.3;3.4)
Men	22/177 (12.4%)			
**Origin**				
Bolivia	127/486 (26.1%)	61.7 (19.5;195.3)	71.2 (22.4;226.4)	31.7 (7.2;139.5)
Other	3/528 (0.6%)			
**Mother with ** ***T. cruzi*** ** infection**	26/78 (33.3%)	5.9 (3.4;10.3)		6.5 (1.9;22.8)
**Mother without ** ***T. cruzi*** ** infection**	47/604 (7.8%)			
**Previous triatomine bite**	35/113 (31.0%)	7.1 (3.9;12.0)		1.8 (0.7;4.6)
**No previous triatomine bite**	20/336 (6.0%)			

°Adjustment for age, sex and origin (patients included in this model: n = 1012).

*Adjustment for all variables in table 2 (patients included in this model: n = 378).

### Clinical description

**Figure 1:**
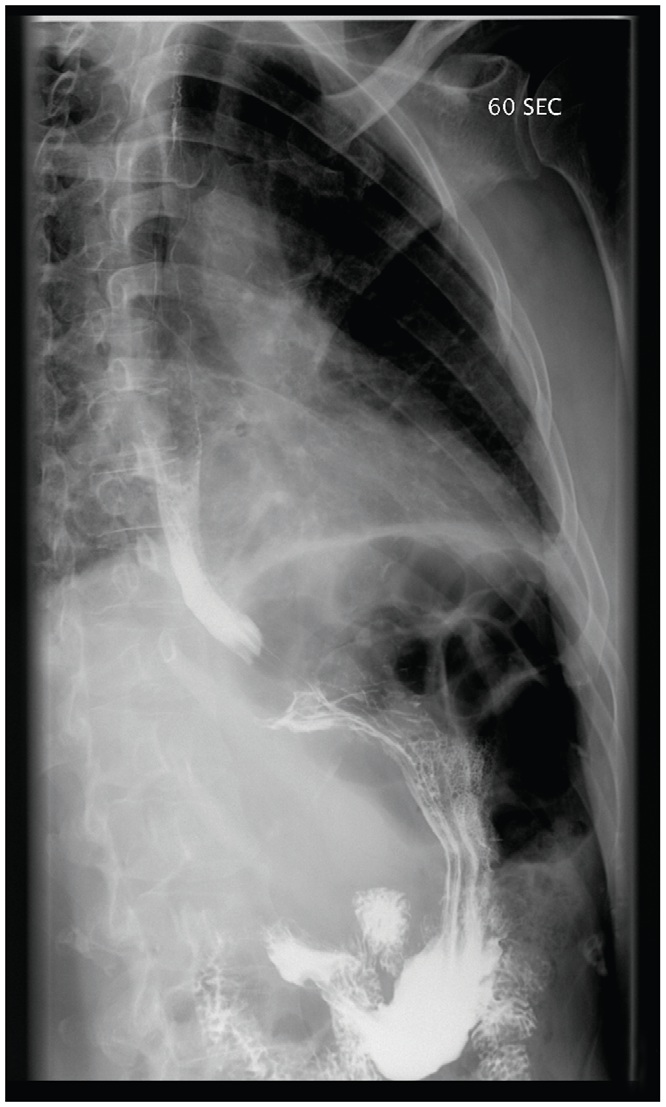
Gastro-oesophageal barium examination in left posterior oblique view in upright position in a *T. cruzi* infected individual showing delayed bolus elimination without oesophageal dilatation (de Rezende stage I) one minute after ingestion.

Clinical evaluation was performed in 124 patients (95.4%), whereas 6 patients were lost to follow-up due to unexpected departure from Switzerland. Out of 14 patients (11.3%) with ECG abnormalities consistent with CCC, 12 (9.7%) were classified as grade A, one as grade B2 (0.8%) and one could not be fully investigated (Table 3). Twelve (9.7%) other patients with normal ECG had symptoms or signs consistent with heart disease. Seven of them underwent further investigations. Four had echocardiographic signs of low-grade diastolic dysfunction and one showed coronary sinus dilatation. Two others presented rhythmic abnormalities on Holter recording. In the absence of definite criteria defined by the Brazilian consensus, we did not consider these seven patients as cases of CCC. Twenty-one (16.9%) patients reported dysphagia (n = 10) and/or severe constipation (n = 16). Barium studies were performed in 16 patients. One patient (0.8%) had grade I oesophageal involvement (Figure 1). 109 (87..9%) patients were classified as chronic infection in the indeterminate form.

**Table 3:** Description and staging of patients with Chagas disease and ECG abnormalities in Geneva, Switzerland.

Patient (sex age)	Symptoms	Signs	ECG	Echocardiogram	Holter	*Staging*
F 28	chest pain	none	inversed T wave avF	normal	PVB (576/24h), bradycardia 45′	*A*
F 35	-	none	partial RBBB	normal	normal	*A*
F 40	palpitation	none	inversed T wave v1-v3	normal	normal	*A*
F 43	-	none	AVB grade I	normal	sustained AT	*A*
F 45	-	none	RBBB	normal	PVB (480/24h), PSVB	*A*
F 45	dyspnea, syncope	none	RBBB	normal	PSVB, sustained AT	*A*
F 46	-	none	bradycardia	normal	PVB (264/24h), PSVB, bradycardia 39′	*A*
F 46	-	none	PSVB, bigeminisme	-	-	*unknown*
F 50	dyspnea, chest pain, palpitation	none	LBBB	LVEF 35%, global hypokinesia	PVB (3960/24h), non-sustained VT	*B2*
F 51	syncope	none	RBBB, bradycardia	normal	bradycardia 34′	*A*
F 54	chest pain, palpitation	none	PVB	diastolic dsyfunction grade 2	PVB (9504/24h)	*A*
F 56	-	none	PVB, LAFB	normal	PVB (28464/24h), bi-trigeminisme	*A*
F 59	-	none	inversed T wave v2-v3, q wave in D1, avL	diastolic dysfunction grade 1, dilated LA	PVB (432/24h), PSVB, bradycardia 44′	*A*
*M 44*	*chest pain*	*none*	*LAFB, inversed T wave avF*	*normal*	*normal*	*A*

RBBB, right bundle branch block; LBBB, left bundle branch block; LAFB, left anterior fascicular block; AVB, atrio-ventricular block; PSVB, premature supra-ventricular beat; PVB, premature ventricular beat; LA, left atria; AT, atrial tachycardia; LVEF, left ventricular ejection fraction.

### Blood and organ donation

Two-hundred forty seven participants (24.4%) and twenty-two patients (16.9%) had already donated blood. Twenty-four (18.5%) and 34 (26.2%) patients expressed willingness to donate blood and organs outside Latin America, respectively (Table 4).

**Table 4:** Frequency of history and intention of blood and organ donation in Latin American migrants living in Geneva, Switzerland.

	Latin American migrants (n = 1012) N (%)	Bolivian migrants (n = 486) N (%)	Migrants with Chagas disease (n = 130) N (%)
**History of blood donation**	247* (24.4)	109 (22.4)	22 (16.9)
in Latin America	208 (84.2)	96 (88.1)	22 (100)
in Europe	17 (6.9)	1 (0.9)	0
not stated	27 (10.9)	13 (11.9)	0
**Intention to give blood outside Latin America**	206 (20.4)	70 (14.4)	24 (18.5)
**History of organ donation**	0	0	0
**Intention to give organ**	360 (35.6)	149 (30.7)	34 (26.2)

*Five individuals have donated blood in more than one geographical region.

## Discussion

We found a high (12.8%) prevalence of Chagas disease among 1012 Latin American migrants attending an urban primary care centre and two Latino churches in Geneva, Switzerland. This figure is much higher than previously reported in Canada (1%) and Germany (2%), but lower than the very high (41%) prevalence found at a referral centre in Spain [5],[22],[23]. Our finding is mostly explained by the high proportion (48%) of Bolivian migrants in the study cohort, of whom 26.2% were diagnosed with Chagas disease. This figure is consistent with recent epidemiological studies conducted in affected provinces of Bolivia (Santa Cruz, Cochabamba), where most of the Bolivian migrants living in Geneva originate from [24],[25]. Bolivian participants were not over-represented in our study as they constitute 42% of Latin American migrants consulting in our primary care facility. Bolivian origin was the main predictive factor for *T. cruzi* infection, in concordance with other reports from non-endemic countries [5],[9]. Only three of the 130 patients originated from other countries (Argentina and Brazil). This is unclear whether these three patients were infected in their country of origin or in Bolivia, where they lived for several years. The absence of cases diagnosed in migrants from other endemic countries is likely to be explained by the insufficient number of persons tested, the possible effect of cluster sampling (as shown in Bolivians) and the lower average national prevalence of *T. cruzi* infection in other Latin American countries [4]. Nevertheless, cases originating from most Central and South American countries have been reported in non-endemic countries [5],[14]. Therefore, consideration for *T. cruzi* infection should not be restricted to Bolivians.

Older migrants were at increased risk for *T. cruzi* infection, most likely due to a longer and more intense exposition to vectorial transmission in their home country. Vector control campaigns have resulted in a sharp reduction of transmission in endemic countries during the last decades, therefore conferring relative protection to younger generations [3]. History of maternal infection (defined by positive serology in the home country) was also a strong and independent predictive factor for infection. Being borne from an infected mother cumulates the risk of vertical transmission and shared exposure to vectorial transmission.

The risk of unrecognized vertical transmission in non-endemic countries is increased by the low proportion of patients aware of being infected, the lack of clinical signs in most infected newborns and the absence of systematic prenatal screening program [1],[9],[10]. Screening should be offered to women of childbearing age, pregnant women and their offspring (in case of proven maternal infection) [9].

Only 4% of patients had valid residency permit and health insurance. It is estimated that several millions of migrants at risk for Chagas disease reside undocumented in Europe and in North America [4],[26]. In many countries, such as Switzerland, undocumented migrants face difficulties to access preventive and curative care. This socio-economic dimension must be taken into consideration by policy makers at the planning stage of screening programs for Chagas disease in non-endemic countries.

ECG abnormalities consistent with CCC were found in 11.3% of cases, a proportion comparable to previous reports [5],[27]. Most patients who could be classified were in grade A CCC according to the Brazilian consensus classification. One patient presented with advanced stage cardiopathy (grade B2) requiring specific therapy. Interestingly, five symptomatic patients with normal ECG had low-grade diastolic dysfunction or coronary sinus dilatation by echocardiography and two others presented rhythmic abnormalities on Holter recording. In the absence of identified alternative aetiology, the diagnosis of early stage CCC is possible. However, the Brazilian criteria do not allow these patients to be classified as such. More studies are needed to define the diagnostic and prognostic value of echocardiography and Holter in symptomatic cases with normal ECG. The low rate of advanced CCC in our study can be explained by the overall young patients' age and, possibly, by the healthy migrant effect. The latter implies that persons who initiate a long distance migration tend to be healthier than persons who do not migrate [28]. Nevertheless, cases of advanced CCC have also been diagnosed in Geneva outside the study period [18],[19]. The disease burden and treatment cost of CCC is recognized as an emerging challenge in some non-endemic countries like the USA and Spain [8],[14]. Only one patient had radiological evidence of low-grade digestive tract alteration consistent with Chagas disease. This low prevalence of digestive tract complications is comparable to findings reported in Spain [5]. As strict clinical criteria were used before undergoing barium studies, we can not exclude to have missed one or more paucisymptomatic case(s).

*T. cruzi* transmission by blood transfusion has been sporadically reported in North America and in Europe [1],[11],[15]. Persistent parasitemia in infected blood donors can lead to infected donations over a long period of time [29]. In our cohort, 24.4% participants and 16.9% of *T. cruzi* infected patients had a prior history of blood donation. Despite a relatively short time (mean: 4.9 years) spent outside Latin America, 6.9% of participants had already donated blood in North America or in Europe. Moreover, a significant proportion of participants and of *T. cruzi* infected patients expressed the intention to donate blood outside Latin America in the future. This positive attitude towards blood donation and the large proportion of patients unaware of being infected highlights the risk of blood-borne transmission and support the implementation of preventive measures in non-endemic countries.

Organ transplant is a rare mode of transmission that has been reported both in endemic and non-endemic countries [30]. Chagas disease can present as a fulminant systemic disease in immunosuppressed patients [1]. In our cohort, none of the participants had donated organs and none of the *T. cruzi* infected patients had a previous history of organ transplant. However, a high proportion of participants and cases considered organ donation while alive or after passing away. Health professionals involved in organ transplantation should be informed or reminded that organ donors or recipients at risk of being infected require screening for Chagas disease.

The high proportions of migrants with no legal registration and of Bolivian origin, as well as the recruitment limited to one city, represent the main limitations of this study as they partially restrict the extrapolation of our findings to other settings. We believe that these limitations are counter-balanced by the large population screened and by the choice of a primary care setting as a recruitment site. Therefore, our study may offer a valuable insight into the current trends of this emerging health problem in Europe.

According to our and others' findings, we recommend screening for Chagas disease in priority all Latin American persons at increased chance of (1) infection (e.g. Bolivian origin, diagnosis of Chagas disease in the mother or in other close family members, prior history of blood transfusion in endemic countries, presence of suggestive cardiac or digestive complaints), (2) severe illness (e.g. immunosuppressed individuals), (3) transmitting *T. cruzi* to others (e.g. pregnant women and women of child bearing age, blood or organ donors), and (4) cure with existing treatments (newborns and children). Cost-effectiveness studies may help to design more rational recommendations. Considering the millions of persons at risk who have recently migrated outside Latin America, medical students and physicians in non-endemic countries must be made aware of the emergence of this neglected tropical disease.

## Supporting Information

Alternative Language Abstract S1 Translation of the abstract into French by YJ.
(0.02 MB DOC)

Checklist S1 STROBE checklist.
(0.09 MB DOC)
