# Supplementary material for: Prevalence, Clinical Staging and Risk for Blood-Borne Transmission of Chagas Disease among Latin American Migrants in Geneva, Switzerland
Source: PLoS Negl Trop Dis. 2010 Feb 2;4(2):e592. doi: 10.1371/journal.pntd.0000592 (PMC2814851; doi:10.1371/journal.pntd.0000592)
Supplement: Alternative Language Abstract S1 — Translation of the abstract into French by YJ. (0.02 MB DOC) [file pntd.0000592.s001.doc]

**Translation of the abstract in French (Yves Jackson)**

**Prévalence, évaluation clinique et risques de transmission sanguine de la maladie de Chagas parmi les migrants latino-américains à Genève, Suisse.**

Fondements :

La migration latino-américaine en direction des Etats-Unis, du Canada et de l’Europe a modifié la distribution de la maladie de Chagas. Pourtant, les données concernant les cas importés et le risque de transmission hors zone endémique restent insuffisantes. Nous avons étudié la prévalence de la maladie de Chagas parmi les migrants latino-américains à Genève, décrit la maladie et évalué les risque de transmission sanguine.

Méthode/résultats

Cette étude observationnelle a inclus des migrants latino-américains consultant dans un centre de santé primaire ou fréquentant deux églises qui ont répondu à un questionnaire et ont été dépistés par deux examens sérologiques (Biomérieux ELISA cruzi™ ; Biokit Bioelisa Chagas™ ). Les personnes infectées ont effectué un bilan médical complet. Les facteurs associés à l’infection ont été analysés par régression logistique uni- et multivariée.

1012 personne ont été dépistées (femmes 83%, âge moyen 37.2 ans (DS 11.3), origine bolivienne 48% (n=485)). 96% résidaient sans permis de séjour en Suisse. La maladie de Chagas a été diagnostiquée chez 130 cas (12.8%; IC95% 10.8-14.9), dont 127 boliviens (26.2%; 95%CI 22.3-30.1). Tous les cas étaient dans la phase chronique. 11.3% présentaient une atteinte cardiaque et 0.8% une atteinte digestive. Les facteurs associés à l’infection étaient l’origine bolivienne (OR 33.2; 95%CI 7.5-147.5), une infection par T. cruzi chez la mère (OR 6.9; 95%CI 1.9-24.3) et un âge supérieur à 35 ans (OR 6.7; 95%CI 2.4-18.8). Alors que 22 personnes parmi les cas (16.9%) avaient déjà donné leur sang, 24 (18.5%) et 34 (26.2%) envisageaient de donné leur sang et respectivement leurs organes hors d’Amérique latine.

Conclusion :

La maladie de Chagas est hautement prévalente parmi les migrants boliviens en Suisse. Le taux de complications cardiaques et digestives est substantiel. Le dépistage des personnes à risque est recommandé, y compris des personnes résidant sans permis de séjour.
